# Anti-Inflammatory Effects of Compounds from Echinoderms

**DOI:** 10.3390/md20110693

**Published:** 2022-11-03

**Authors:** Hardik Ghelani, Md Khursheed, Thomas Edward Adrian, Reem Kais Jan

**Affiliations:** College of Medicine, Mohammed Bin Rashid University of Medicine and Health Sciences, Dubai P.O. Box 505055, United Arab Emirates

**Keywords:** anti-inflammatory activity, inflammatory pathways, marine drugs, echinoderm, sea cucumber, sea urchin, starfish

## Abstract

Chronic inflammation can extensively burden a healthcare system. Several synthetic anti-inflammatory drugs are currently available in clinical practice, but each has its own side effect profile. The planet is gifted with vast and diverse oceans, which provide a treasure of bioactive compounds, the chemical structures of which may provide valuable pharmaceutical agents. Marine organisms contain a variety of bioactive compounds, some of which have anti-inflammatory activity and have received considerable attention from the scientific community for the development of anti-inflammatory drugs. This review describes such bioactive compounds, as well as crude extracts (published during 2010–2022) from echinoderms: namely, sea cucumbers, sea urchins, and starfish. Moreover, we also include their chemical structures, evaluation models, and anti-inflammatory activities, including the molecular mechanism(s) of these compounds. This paper also highlights the potential applications of those marine-derived compounds in the pharmaceutical industry to develop leads for the clinical pipeline. In conclusion, this review can serve as a well-documented reference for the research progress on the development of potential anti-inflammatory drugs from echinoderms against various chronic inflammatory conditions.

## 1. Introduction

Inflammation is an innate immune response to a variety of stimuli, such as infections and tissue injury. The onset of inflammation is characterized by the secretion of several types of chemokines, including cytokines and chemoattractants, which draw leukocytes to the site of injury or infection through the process of extravasation. The immune function of inflammation is mediated by several classes of soluble antimicrobial peptides, including defensins, cathelicidins, marginins, and C-reactive proteins (CRP), and immune cells, including neutrophils, macrophages, natural killer cells, and dendritic cells. All these components of inflammation combine and stimulate opsonization and phagocytosis to clear infections. Phagocytosis and opsonization are induced by several classes of reactive oxygen species (ROS) or reactive nitrogen species induced in neutrophils by the activation of specific signaling pathways [1,2,3]. The process of acute inflammation is necessary to fight infection and tissue injury. However, there are several physiological conditions in which the process of inflammation is persistently stimulated, leading to chronic disease [4,5]. Immune-mediated inflammatory diseases are diverse and manifest in several conditions, such as asthma, rheumatoid arthritis, ulcerative colitis, and Crohn’s disease [6,7].

### 1.1. Inflammatory Pathways and Models 

Multiple inflammatory pathways play a role in innate immunity and activate adaptive immunity to combat the cause of inflammation. These pathways are initiated by several classes of receptors present on leukocytes known as pattern recognition receptors. Common examples of such receptors are (1) the toll-like receptor family (TLR), (2) C-type lectin receptors, (3) retinoic acid-inducible gene-I-like receptors, and (4) nucleotide-binding and oligomerization domain (NOD)-like receptors (NLR) [8,9]. The activation of immune cells such as macrophages, neutrophils, and other immune cells leads to the secretion of cytokines, which sustain the inflammatory response. These cytokines bind to the immune cells and activate their function. The common cytokine receptor families are the: (1) immunoglobulin superfamily, (2) class I cytokine receptor family, (3) class II cytokine receptor family, (4) tissue necrosis factor (TNF) receptor superfamily, and (5) chemokine receptor family. Ligand binding on the pattern recognition receptors or cytokine receptors activates several signaling pathways, which ultimately induces the transcription of several inflammation regulatory genes. There are four broad categories of signaling pathways activated during the inflammation process: (1) the mitogen-activated protein kinase (MAPK) pathway, (2) phosphoinositide 3-kinase signaling pathway, (3) Janus kinase (JAK) signal transducer and activator of transcription (STAT), and (4) I kappa B kinase (IκB)/nuclear factor kappa B (NF-κB) signaling pathways [10,11].

The sustained activation of these signaling pathways underlies the cause of several inflammatory diseases. For instance, the NF-kB signaling pathway is a classic pathway in the regulation of inflammation. The activation of NF-kB via IκBα increases the expression of various downstream inflammatory mediators, such as proinflammatory cytokines (interleukin 1β (IL-1β), IL-6, and TNFα); key proinflammatory enzymes, including inducible nitric oxide synthase (iNOS) and cyclooxygenase-2 (COX-2); and their derivatives nitric oxide (NO) and prostaglandin E2 (PGE2) [12,13]. Multiple experimental models are available to study the activation of inflammatory signaling and transcription for various inflammatory diseases. These experimental models are also widely used to evaluate potential anti-inflammatory compounds and to understand the mechanism(s) of their therapeutic effects. Various experimental models have been designed and implemented to study the preliminary efficacy of anti-inflammatory compounds. For example, carrageenan-induced paw edema in mouse [14] and 12-O-tetradecanoylphorbol-13-acetate (TPA) mouse ear inflammation models [15]. Other specific experimental models are also available and have been used for the assessment of chronic inflammatory diseases, including the dextran sodium sulfate (DSS)-induced colitis model [16], which has been widely used to screen the anti-inflammatory effects of marine drugs. For example, this model was recently used to study the anti-inflammatory effects of polysaccharides isolated from the mussel *Mytilus couscous* [17]. Another well-known model to study cytokine-mediated inflammatory signaling pathways is TNFα-induced intestinal inflammation in colon cancer cell lines [18]. For example, krill oil was screened for its anti-inflammatory effects by using this model in HT-29 and Caco-2 cells [19]. The free fatty acid (FFA)-mediated activation of inflammatory signaling in hepatocytes is a well-known model for nonalcoholic steatohepatitis [20]. Jiena et al. [21] demonstrated that fucoxanthin, a popular marine-derived compound, attenuated FFA-induced inflammation via the AMP-activated protein kinase/nuclear factor erythroid 2–related factor 2/TLR4 signaling pathway in normal human Chang liver cells.

### 1.2. Marine-Derived Anti-Inflammatory Drugs 

Chronic inflammatory conditions pose a major burden on our healthcare system, despite the availability of several synthetic compounds used for the management of these conditions. Over the past decade, the research and development of model systems and evaluation of the efficacy of various compounds have led to the identification of several anti-inflammatory compounds from natural origins [22,23,24]. Marine sources produce a vastly diverse range of bioactive compounds, several of which possess anti-inflammatory potential. Indeed, anti-inflammatory compounds have been derived from marine microorganisms such as seaweeds, corals, and algae [25]. These fall into several classes of bioactive compounds with therapeutic potential for several chronic inflammatory conditions. For example, marine alkaloids from a diverse range of marine organisms have been evaluated for their potential anti-inflammatory activity [26]. Another class of marine compounds act as inhibitors of NF-κB, a mediator that is activated in the inflammation process [27]. Pigments from various marine organisms have been shown to have anti-inflammatory activity and can be used in the management of chronic inflammation [28,29]. For instance, Echinochrome A (EchA, a pigment isolated from sea urchin), briaviodiol A (a cembranoid from a soft coral), and cucumarioside A2 (a triterpene glycoside from sea cucumbers) have been shown to suppress inflammation via the reprogramming of macrophages from M1 to M2 [30]. Seaweeds are classically used as food supplements and have great potential as a source of anti-inflammatory compounds [31,32]. Overall, because of the diversity of classes of bioactive compounds from marine sources with potential applications as anti-inflammatory agents, there is a need to comprehensively catalogue these resources. 

Over the past few decades, attempts have been made to isolate and purify biologically active compounds with potent anti-inflammatory activity from different marine sources. However, very few compounds have been selected for clinical trials and even fewer have reached the market. Despite this low success rate, the hunt for new anti-inflammatory compounds from the diverse marine environment continues. Recently, Li et al. reviewed the anti-inflammatory metabolites from marine organisms such as sponges and corals but did not include larger organisms such as sea cucumbers, sea urchins, and starfish [25]. In this review article, we describe promising anti-inflammatory compounds and crude extracts isolated from echinoderms such as sea cucumbers, sea urchins, and starfish and review their potential molecular mechanisms of action in an effort to shed light on the current state of the research on anti-inflammatory compounds from echinoderms. 

## 2. Methods

During the period between December 2021 and June 2022, separate database searches were conducted on PubMed, Scopus, Web of Science, American Chemical Society (ACS), MDPI, Elsevier, and SpringerLink using various relevant keywords and combinations of keywords, such as “Sea cucumber” + “Anti inflammatory”, “Sea urchin” + “Anti inflammatory”, and “Starfish” + “Anti inflammatory”. The inclusion criteria encompassed only original research articles published in the English language between 2010 and 2022. Editorials, review articles, and any duplicated publications were excluded. As a final step in the screening process, we included only studies thoroughly aligned with the theme of this review. For easier readership and referencing, the review is divided into sections according to the three types of echinoderms and subsections describing the anti-inflammatory activities of the various compounds that originate from them. 

## 3. Anti-Inflammatory Compounds from Sea Cucumbers

Sea cucumbers belong to the class of Holothuroidea and the phylum of Echinodermata. They are globally found in deep seas in benthic areas. Sea cucumbers are harvested for food and are widely consumed in China, Korea, Japan, Malaysia, Indonesia, and Russia. There are approximately 1500 species of sea cucumbers, of which approximately 100 are known for human consumption [33]. Sea cucumbers have received particular attention for their potential therapeutic benefits owing to the availability of a variety of active compounds originating from them that possess medicinal properties [33]. Sea cucumbers are a rich source of bioactive polysaccharides, terpenoids, peptides, lipids, and fatty acids. As a result, sea cucumbers are used as a tonic food and folk medicine in Eastern Asia to cure numerous ailments. East Asian consumers consider sea cucumbers as one of the most luxurious and nutritious foods and use them as a traditional remedy for hypertension, rheumatism, asthma, cuts and burns, joint pain, back pain, wound injuries, kidney problems, reproductive disorders, constipation, and cancer [34,35]. The bioactive substances derived from various species of sea cucumbers and their proposed mechanisms of anti-inflammatory activity are described in the below sections and are summarized in Table 1.

### 3.1. Anti-Inflammatory Activity of Polysaccharides from Sea Cucumbers

**Figure 1:**
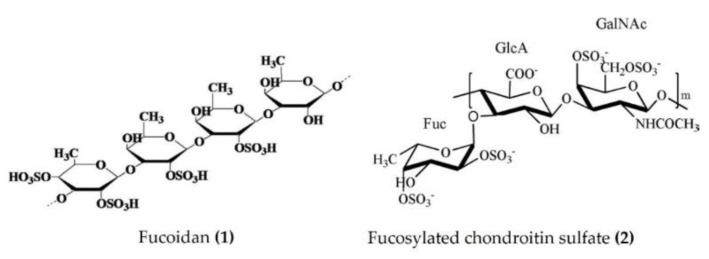
Structures of anti-inflammatory polysaccharides derived from sea cucumbers (structure (**1**) re-used with permission from reference [36], Elsevier, 2016; structure (**2**) re-used with permission from reference [14], Elsevier, 2018).

Several studies have emphasized the valuable anti-inflammatory effects of polysaccharides obtained from sea cucumbers. Sulphated fucan, fucoidan, and fucosylated chondroitin sulfate (FCS) are major polysaccharides isolated from several species of sea cucumbers, such as *Thelenota ananas*, *Stichopus variegatus*, *Holothuria nobilis*, *Ypsilothuria bitentaculata*, *Cucumaria frondosa*, *Stichopus (Apostichopus) japonicus*, *Stichopus choloronotus*, and *Isostichopus badionotus*. These polysaccharides have shown potent anti-inflammatory activity in various cellular, as well as animal, models of chronic inflammation. The chronic administration of fucoidan (Figure 1: (**1**)) derived from *Isostichopus badionotus* reduced the hepatic expression and serum concentrations of inflammatory cytokines and other inflammatory markers (TNFα, IL-1β, IL-6, IL-10, macrophage inflammatory protein 1 (MIP-1), and CRP) in diet-induced obese mice. The anti-inflammatory response of fucoidan was achieved by the inactivation of JNK and IκB/NF-κB pathways in hepatocytes [36]. Moreover, fucoidan isolated from *Apostichopus japonicus* reduced the expression of TNFα, IL-1β, and IL-6 by inactivating the MAPK/ NF-κB pathway in the lipopolysaccharide (LPS)-challenged liver injury mouse model [37]. Fucoidan, isolated from *Acaudina molpadioides*, alleviated renal fibrosis and inflammation by decreasing the expression of transforming growth factor β1 (TGFβ1), plasminogen activator inhibitor 1, and phosphorylated Smad3 in diabetic mice [38]. Fucoidans derived from *Thelenota ananas* prevented ethanol-induced gastric ulceration by downregulating the expression of proinflammatory cytokines and related transcription factors (TNFα, IL-6, and NF-κB) [39]. Fucoidan oligosaccharides isolated from *Pearsonothuria graeffei* and *Isostichopus Badionotus* alleviated high-fat diet (HFD)-induced low-grade inflammation by lowering the serum TNFα and LPS levels in mice [40]. The primary glycosaminoglycan FCS was purified from *Isostichopus badionotus* and screened in vivo and in vitro for anti-inflammatory activity. FCS downregulated the NF-ĸB gene expression and thereby suppressed the expression of downstream genes such as COX-2, iNOS, and TNFα and attenuated the inflammation and tissue damage caused by TPA in a mouse ear inflammation model [15]. Furthermore, FCS (Figure 1: (**2**)) isolated from edible sea cucumbers *Apostichopus japonicus*, *Stichopus chloronotus*, *Cucumaria djakonovi*, and *Acaudina molpadioidea* reduced carrageenan-induced paw edema in a mouse model [14,41]. More recently, Zhu et al. [42] demonstrated that the sulfated fucan/FCS-dominated polysaccharide fraction from low-edible-value sea cucumber species reduced the levels of proinflammatory cytokines (such as TNFα and IL-6) of HFD and streptozotocin (STZ)-induced type 2 diabetic rats, indicating a decreased inflammatory response. FCS, isolated from *Lymantria grisea*, inhibited neutrophil recruitment and TNFα production in thioglycollate-induced peritonitis and LPS-induced lung inflammation mouse models [43]. Interestingly, the heteroglycan (sulphated polysaccharide) fractions derived from the cartilage of *Curcumaria frondosa* (at concentrations of 0.1–100 µg/mL) increased the oxidative stress and decreased cell viability, as evidenced by the induced levels of TNFα, IL-6, and IL-10 in THP-1 macrophages [44]. 

### 3.2. Anti-Inflammatory Activity of Triterpenoid Glycosides from Sea Cucumbers

**Figure 2:**
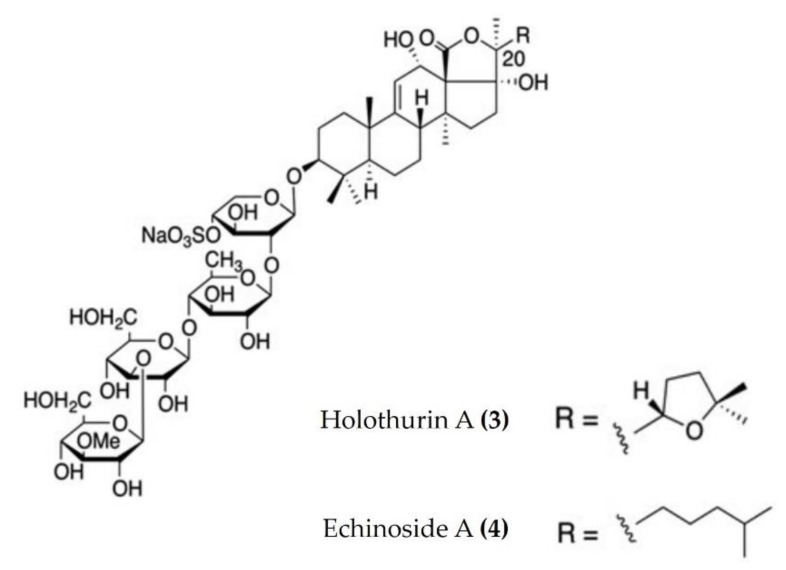
Structures of anti-inflammatory triterpenoid glycosides derived from sea cucumbers (structures (**3**) and (**4**) re-used from reference [47]).

Triterpenoid glycosides are broadly distributed in plants, animals, and marine organisms such as holothurians and sponges. Triterpenoid glycosides play a crucial role in chemical defenses and possess a variety of pharmacological activities. Approximately 300 triterpenoid glycosides have been identified and categorized from sea cucumbers. Relatively few anti-inflammatory triterpenoid glycosides from sea cucumbers are documented in the literature. Triterpenoid glycosides isolated from the Egyptian sea cucumber *Holothuria thomasi* significantly decreased the serum levels of TNFα and IL-6, as well as glucose, adiponectin, liver malondialdehyde, and α-amylase activity, in STZ-induced diabetic rats [45]. Similarly, a liposomal preparation of triterpenoid glycoside (Holothurin A (Figure 2: (**3**)) and Echinoside A (Figure 2: (**4**)) isolated from *Pearsonothuria graeffei* reduced the inflammation by inhibiting the release of proinflammatory cytokines and infiltration of macrophages in the adipose tissue of HFD-fed obese mice. Moreover, this liposomal triterpenoid glycoside preparation significantly reduced the PGE2 levels in adipose tissue by modulating the p-ERK/cPLA2/COX-1 pathway [46]. Moreover, holothurin A and echinoside A also attenuated inflammation by downregulating the expression of proinflammatory cytokines in vascular and peritoneal macrophages of ApoE^−/−^ mice [47].

### 3.3. Anti-Inflammatory Activity of Peptides from Sea Cucumbers 

**Figure 3:**
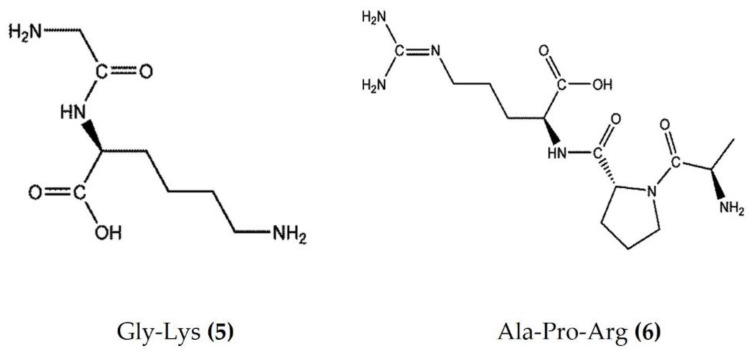
Structures of anti-inflammatory peptides derived from sea cucumbers (structures (**5**) and (**6**) re-used with permission from reference [50], John Wiley and Sons, 2021).

Marine bioactive peptides are short amino acid sequences, generally 2–20 amino acids in length, that are biologically inactive within their respective precursor proteins until released by enzymic hydrolysis. Hydrolysates derived from marine sources have attracted considerable interest within the scientific community due to their diverse biological activities and applications in clinical treatment [48]. Protein constitutes more than 70% of the sea cucumber body and is an effective source of food-borne bioactive peptides. The enzymatic hydrolysates extracted from *Apostichopus japonicus* and *Acaudina leucoprocta* exhibited potent anti-inflammatory activity in a diet-induced hyperuricemic renal inflammation mouse model. The hydrolysates downregulated the proinflammatory cytokines (TNFα, IL-1β, and IL-6) and upregulated the anti-inflammatory cytokines TGFβ and IL-10 by modification of the TLR4/myeloid differentiation primary response 88(MyD88)/NF-κB signaling pathway. The amino acid sequences of peptides found in hydrolysates of *Apostichopus japonicus* and *Acaudina leucoprocta* have been characterized by MALDI-TOF/TOF-MS. GPSGRP (Gly-Pro-Ser-Gly-Arg-Pro) and GPAGPR (Gly-Pro-Ala-Gly-Pro-Arg) were identified as the two major anti-inflammatory peptides from *Apostichopus japonicus*, while PQGETGA (Pro-Gln-Gly-Glu-Thr-Gly-Ala) and GFDGPEGPR (Gly-Phe-Asp-Gly-Pro-Glu-Gly-Pro-Arg) were detected with the highest abundance in *Acaudina leucoprocta* [49]. Zhang et al. [50] also reported two peptides (Gly-Lys (Figure 3: (**5**)) and Ala-Pro-Arg (Figure 3: (**6**))) from *Apostichopus japonicus* that showed marked anti-inflammatory activity in a CuSO4-induced zebrafish inflammation model. Moreover, a molecular docking analysis revealed that both peptides have a high affinity to bind and inhibit angiotensin-I converting enzyme (ACE-1), a therapeutic target in the treatment of inflammatory conditions [50]. Low molecular weight sea cucumber peptides (SCP, rich in aspartic acid, glycine, proline, and glutamic acid) isolated from *Stichopus japonicus*, a sea cucumber widely distributed along the coasts of China and Japan, displayed potent anti-inflammatory activity in LPS-stimulated RAW264.7 murine macrophages by the inhibition of NF-κB and activation of MAPK in macrophages [51]. Moreover, the anti-inflammatory activity of another SCP (rich in glycine, glutamic acid, and proline) isolated from *Stichopus japonicus* was also demonstrated in vivo, where it significantly inhibited serum proinflammatory cytokines and downregulated the overexpression of TLR4 and NF-κB in gastrocnemius muscles of rats [52]. The hydrolysate bioactive fraction, isolated from the sea cucumber species *Holothuria forskali*, reduced the vascular cell adhesion molecule (VCAM)-1 and IL-6 expression levels in endothelial cells and intercellular adhesion molecule (ICAM)-1 expression in subcutaneous adipose tissue and was shown to inhibit ACE-1 enzyme activity in an in vitro assay [53]. Recently, Jo et al. [54] isolated sea cucumber extracellular matrices (body wall collagen) from *Stichopus japonicus*, which possessed potent anti-inflammatory activity in a TNFα and IL-1β-induced osteoarthritis in vitro model. Moreover, the major yolk protein isolated from *Stichopus japonicus* attenuated experimental DSS-induced colitis by preventing tissue damage, promoting the expression of anti-inflammatory cytokines, and increasing the levels of short-chain fatty acids [55]. Similarly, sea cucumber (*Stichopus japonicus*) enzymatic hydrolysates have been shown to alleviate the inflammatory response via the downregulation of RANKL (receptor activator of NF-kB) and thereby inhibiting the NF-kB pathway in ovariectomized rats [56]. 

### 3.4. Anti-Inflammatory Activity of Lipids and Fatty Acids from Sea Cucumbers

**Figure 4:**
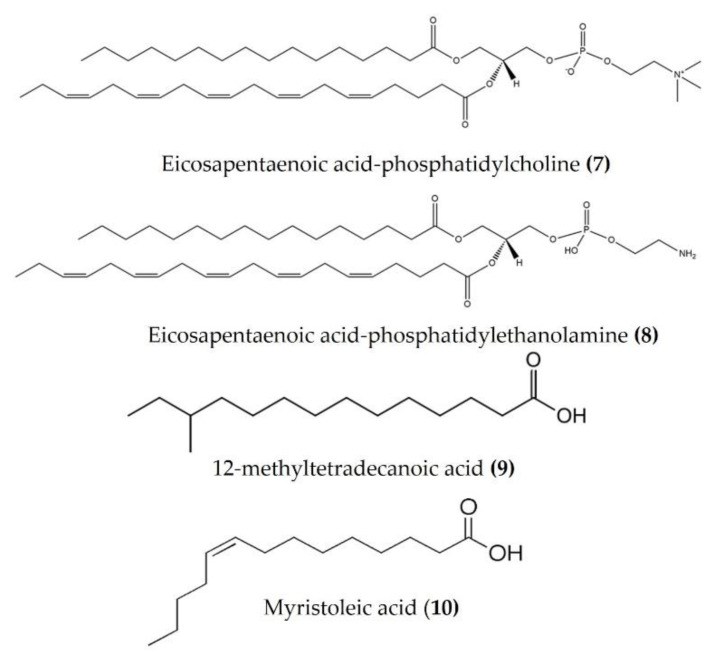
Structures of anti-inflammatory fatty acid derivatives derived from sea cucumbers (structures (**7**) and (**8**) re-used from reference [59]).

Lipids are best known for their integral role in biological membranes and as signaling molecules in the cytoplasm. Sea cucumbers are rich sources of lipids, phospholipids, and various fatty acids that exert a wide variety of biological activities. Eicosapentaenoic acid (EPA), isolated from *Cucumaria frondosa*, has a potent anti-obesity effect and modulates the peroxisome proliferator activated receptor γ (PPARγ) signaling in the inflammatory condition of insulin resistance, as well as type 2 diabetes [57,58]. Phosphatidylcholine (EPA-PC) (Figure 4: (**7**)) and phosphatidylethanolamine (EPA-PE) (Figure 4: (**8**)) from *Cucumaria frondosa* improve chronic inflammation and alter the interaction between macrophages and adipocytes [59,60]. Moreover, EPA-PC and EPA-PE diminish chronic inflammation by promoting the M2-dominant polarization of macrophages in white adipose tissue, as observed in 3T3L1 and RAW264.7 transwell coculture. EPA-PC and EPA-PE also inhibit the transactivation of NF-κB in RAW264.7 macrophages and upregulate PPARγ expression in 3T3-L1 adipocytes in the coculture, indicating that they may alleviate adipose tissue inflammation [59,60]. Both EPA-PC and EPA-PE reduced the serum TNFα, IL-6, and monocyte chemoattractant protein (MCP), increased the serum heme oxygenase-1 (HO-1) levels (one of the most abundant enzymes involved in oxidative stress and with anti-inflammatory properties), and attenuated macrophage infiltration in the liver and adipose tissue of high-fat high-sucrose diet-induced inflammation in mice [61]. Moreover, EPA-PC inhibited amyloid β-protein-induced neurotoxicity by alleviating the NLR family pyrin domain-containing 3 (NLRP3) inflammasome in an Alzheimer’s disease rat model [62]. EPA phospholipids derived from *Cucumaria frondosa* mitigated obesity-induced inflammation by reducing TNFα and IL-6 in the serum of diet-induced obese mice [63]. A fatty acid-rich fraction (n-hexane phase) of *Apostichopus japonicus* has shown several immunomodulatory activities in an ovalbumin-induced allergic airway inflammation mouse model and in splenocytes [64]. This fraction reduced eosinophil infiltration and goblet cell hyperplasia and attenuated IL-4, IL-5, IL-13, and IL-17 in the spleen and bronchoalveolar lavage fluid of mice. It also increased the expression of anti-inflammatory cytokines (TGFβ and IL-10) in bronchoalveolar lavage fluid and a splenocyte culture medium [64]. Frondanol^TM^ is a nutraceutical lipid extract (rich in 12-methyltetradecanoic acid (Figure 4: (**9**)) and myristoleic acid (Figure 4: (**10**))) of the intestines of the edible Atlantic sea cucumber *Cucumaria frondosa*. Frondanol has potent anti-inflammatory activity and has been shown to attenuate inflammation in an adjuvant arthritis rat model, as well as an ear edema mouse model [65]. It also potently inhibits lipoxygenase (LOX) pathways, reducing leukotriene production in human polymorphonuclear cells [65]. More recently, Subramanya et al. [16] demonstrated that chronic treatment with Frondanol decreased inflammation in a DSS-induced colitis mouse model. Frondanol markedly reduced proinflammatory cytokine mRNA expression in colon tissue and cytokine levels in the circulation, while inhibiting production of the proinflammatory mediator leukotriene B4 (LTB4) [16]. Sphingolipids isolated from *Cucumaria frondosa* decrease the serum proinflammatory cytokines, as well as mRNA expression in the adipose tissue of obese mice by the inhibition of phosphorylated JNK, IκB, and NF-κB nuclear translocation [66]. Furthermore, these sphingolipids attenuated renal fibrosis and inflammation via the inactivation of TGFβ/Smad signaling pathway in STZ-HFD-fed type 2 diabetic mice [67]. The cerebrosides and glucosylceramides extracted from *Cucumaria frondosa* reduced the expression of proinflammatory cytokines and thereby improved the insulin sensitivity in adipose tissues of high-fructose diet-fed rats [68]. 

### 3.5. Anti-Inflammatory Activity of Miscellaneous Crude Extracts of Sea Cucumbers

Several aqueous and organic solvent-extracted fractions of various sea cucumber species have shown marked anti-inflammatory activity in various in vivo and in vitro models. Frondanol A5 (isopropyl alcohol/water extract of epithelium from the sea cucumber *Cucumaria frondosa*) decreased the production of inflammatory cytokines, such as IL-1α, IL-1β, IL-2, IL-4, IL-6, IL-10, IL-12, IL-17A, interferon gamma (IFNγ), and TNFα, in an APC^Min/+^ mouse model [69]. Additionally, Frondanol A5 also suppressed the mRNA expression of inflammatory markers (5-LOX and 5-lipoxygenase activating protein (FLAP)) and an angiogenesis marker in intestinal tumors [69]. An ethyl-acetate fraction (which contains mostly phenolic compounds) of the sea cucumber *Holothuria scabra* attenuates inflammation in vitro by inhibiting the production of NO and proinflammatory cytokines via the NF-κB and JNK pathways [70]. The ethyl-acetate fraction from another sea cucumber species, *Stichopus japonicus*, markedly reduced inflammation by inhibiting the production of NO and PGE2 (via downregulating the iNOS and COX-2 gene expression). Moreover, the fraction was shown to suppress the transcription of proinflammatory cytokines in LPS-stimulated murine macrophages through suppression of the phosphorylation of MAPK [71]. An aqueous fraction of *Stichopus japonicus* reduced production of the proinflammatory cytokines IL-6, and TNFα in LPS-stimulated macrophages and inhibited antigen-induced mast cell degranulation and IL-4 mRNA expression in antigen-stimulated RBL-2H3 rat basophil [72]. An aqueous extract of the sea cucumber *Stichopus chloronotus* demonstrated both anti-inflammatory and antioxidative activities by upregulating cartilage-specific markers such as collagen type II, aggrecan core protein, and SRY-Box transcription factor 9 (sox-9) expression and downregulating collagen type 1, IL-1, IL-6, IL-8, matrix metalloproteinases (MMP)-1, MMP-3, MMP-13, COX-2, iNOS, and protease-activated receptor 2 (PAR-2) expression [73]. An aqueous extract of *Holothuria polii* attenuated the levels of the inflammatory markers IL-6, NO, and MMP-9 in mouse mammary epithelial SCp2 cells and the levels of IL-1β produced in THP-1 human monocytes [74]. A methanol body wall extract of the sea cucumber *Holothuria atra* downregulated the proinflammatory cytokines TNFα, and IL-1β in a cecal ligation and puncture rat model [75]. The body wall preparation of *Isostichopus badionotus* suppressed the expression of proinflammatory genes, including TNFα, iNOS, COX-2, NF-κB, and IL-6, in a mouse ear inflammation model [76]. Ethanol extracts obtained from four species of sea cucumbers belonging to the family Holothuriidae (namely, Holothuriidae ni1, Holothuriidae ni2, Holothuriidae ni3, and Holothuriidae ni4) showed potent antioxidant and COX-2 inhibitory activities in vitro [77].

**Table 1:** Anti-inflammatory bioactive compounds and extracts derived from sea cucumbers.

Species	Bioactive Compounds/Extracts	Model	Mechanism of Anti-Inflammatory Activity	Ref.
*Apostichopus* *japonicus* and *Stichopus* *chloronotus*	Fucosylated chondroitin sulfate	Carrageenan-induced paw edema in rats	Reduces neutrophil migration, decreases paw edema	[14]
*Isostichopus* *badionotus*	Fucosylated chondroitin sulfate	TPA-induced ear inflammation in mice	Suppresses TPA-mediated up-regulation of TNFα, IL-6, NF-ĸB, iNOS, IL-10, IL-11, COX-2 and STAT3 genes in mouse ear tissue	[15]
*Isostichopus* *badionotus*	Fucoidan	High-fat high-sucrose diet induced obese mouse model	Regulates serum inflammatory cytokines (TNFα, CRP, MIP-1, IL-1β, IL-6, and IL-10) and their mRNA expression, inactivates JNK and IκB/NF-κB pathways	[36]
*Holothuria* *albiventer* and *Cucumaria frondosa*	Sulfated fucan /FCS	HFD and STZ-induced type 2 diabetes mellitus model	Suppresses production of proinflammatory cytokines (TNFα and IL-6)	[42]
*Holothuria thomasi*	Triterpenoid Glycoside	STZ-induced diabetic rats	Decreases serum IL-6, TNFα levels	[45]
*Pearsonothuria graeffei*	Triterpenoid glycoside liposomes	HFD-fed obese mice	Reduces TNFα, IL-1β, and IL-6 and infiltration of macrophages in obese mice via p-ERK/cPLA2/COX-1 pathway and reduces the PGE2 levels	[46]
*Apostichopus* *japonicus* and *Acaudina leucoprocta*	Small peptides (GPSGRP, GPAGPR, PQGETGA, GFDGPEGPR)	Diet-induced renal inflammation in mice	Downregulates the transcription of proinflammatory cytokines, upregulates anti-inflammatory cytokines, and inhibits TLR4/MyD88/NF-κB signaling pathway	[49]
*Apostichopus* *japonicus*	Peptide (GL, APA)	CuSO4-induced neuromast damage in zebrafish model	Suppresses leukocyte migration, ACE enzyme inhibition	[50]
*Stichopus* *japonicus*	Peptides	LPS-stimulated RAW264.7 macrophages	Suppresses NO production and mRNA expression of inflammatory mediators (iNOS, TNFα, IL-1β and IL-6) through inhibition of NF-κB and MAPK signaling pathways	[51]
*Stichopus* *japonicus*	Peptides	Endurance swimming rat model	Reduces inflammation by suppression of TLR4 expression and NF-κB activation in gastrocnemius muscle tissue of rat	[52]
*Holothuria forskali* and *Parastichopus tremulus*	Hydrolysate	In vitro assay of ACE-1, human umbilical endothelial and Caco-2 cells co-culture	Reduces VCAM-1, ICAM-1 and IL-6 expression in endothelial cells, inhibits ACE-1	[53]
*Stichopus* *japonicus*	Collagen	Synoviocytes osteoarthritis model	Suppresses mRNA expression of inflammatory cytokines in synoviocytes	[54]
*Stichopus* *japonicus*	Major yolk protein from body wall	DSS-induced colitis in mice	Prevents tissue damage, promotes IL-4 and IL-10, increases short-chain fatty acids	[55]
*Apostichopus* *japonicus*	Body wall hydrolysate	Ovariectomized-induced osteoporosis in rat	Blocks NF-kB activation by downregulating RANKL, suppresses proinflammatory cytokines	[56]
*Cucumaria* *frondosa*	Eicosapentaenoic acids	LPS-stimulated RAW264.7 macrophages and 3T3-L1 adipocytes, high-fat high-sucrose diet-induced inflammatory mouse model	Reduces elevated levels of serum TNFα, IL-6 and MCP-1, attenuates macrophage infiltration in the liver in mice, attenuates the phosphorylation of NF-κB in Raw264.7 macrophages and increased PPARγ expression in 3T3-L1 adipocytes	[60]
*Cucumaria* *frondosa*	Frondanol	DSS-induced colitis in mice	Reduces inflammation-associated changes in colon in mice, reduces proinflammatory cytokine content at the protein and mRNA level, reduces proinflammatory LTB4 levels	[16]
*Apostichopus* *japonicus*	Fatty acids	Allergic airway inflammation mouse model and in splenocytes	Reduces eosinophil infiltration and goblet cell hyperplasia, attenuates IL-4, IL-5, IL-13, IL-17 and increases level of anti-inflammatory cytokines TGFβ and IL-10	[64]
*Cucumaria* *frondosa*,	Sphingolipids	High-fat high-fructose diet-induced obese mice	Decreases serum proinflammatory cytokines IL-1β, IL-6 and TNFα, increases anti-inflammatory IL-10, via inhibition of phosphorylation of JNK and translocation of NF-κB	[66]
*Cucumaria* *frondosa*	Frondanol A5	APC^Min/+^ mouse model	Attenuates circulating inflammatory cytokines and suppresses mRNA expression of inflammatory markers such as 5-LOX and FLAP	[69]
*Holothuria* *scabra*	Ethyl acetate Extract	LPS-stimulated RAW264.7 macrophages	Inhibits proinflammatory cytokines mRNA and protein, suppresses NO production via inhibition of iNOS, down-regulates IκB/NF-κB and JNK expression in macrophages	[70]
*Stichopus* *japonicus*	Ethyl acetate fraction	LPS-stimulated RAW264.7 macrophages	Inhibits proinflammatory cytokines via suppression of the phosphorylation of MAPK, ERK and p38 MAPK signaling pathway	[71]
*Stichopus* *japonicus*	Aqueous Fraction	LPS-stimulated RAW264.7 macrophages and antigen-stimulated RBL-2H3 rat basophil.	Reduces proinflammatory cytokines IL-6 and TNFα, inhibits antigen-induced mast cell degranulation and IL-4 mRNA expression	[72]
*Stichopus* *chloronotus*	Aqueous Extract	Osteoarthritis-articular cartilage model	Upregulates cartilage specific markers, downregulates IL-1β, IL-6, IL-8, MMP-1, MMP-3, MMP-13, COX-2, iNOS and PAR-2 expression, increases glycosaminoglycans and reduces NO and PGE2 production	[73]
*Holothuria* *polii*	Aqueous Extract	TPA-activated THP-1 cells and endotoxin-induced mammary epithelial SCp2 cells	Decreases levels of inflammatory markers IL-6, NO and MMP-9 in the mouse mammary SCp2 cells, decreases the level of IL-1β in THP1 cells	[74]

## 4. Anti-Inflammatory Compounds from Sea Urchins 

Sea urchins are seafloor-dwelling invertebrates belonging to the phylum Echinodermata that have high nutritional and medicinal properties. They are rich in vitamins, minerals, proteins, fatty acids, and polysaccharides and possess anticancer, anticoagulant/antithrombotic, antimicrobial, anti-inflammatory, and antioxidant properties. The extracts and hydrolysates of sea urchins contain various bioactive compounds, especially glycosides, pigments, sphingolipids, glycolipids, sulphate, and phospholipids [78]. The anti-inflammatory properties of various active components isolated from sea urchins are summarized in Table 2.

### 4.1. Anti-Inflammatory Activity of Pigments from Sea Urchins

**Figure 5:**
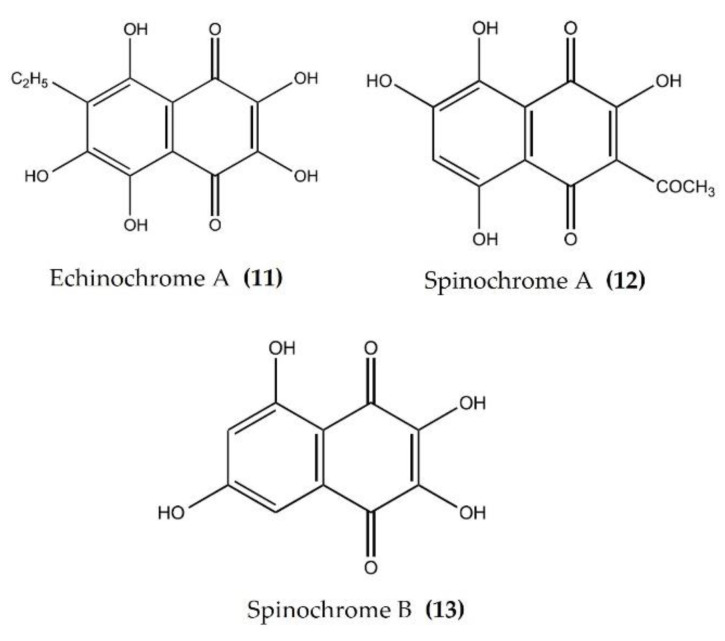
Structures of anti-inflammatory pigments derived from sea urchins (structures (**11**–**13**) re-used with permission from reference [85], Elsevier, 2020).

Pigments isolated from the shells and spines of sea urchins are currently being widely studied for biological activity. EchA (Figure 5: (**11**)) is widely distributed in various species of sea urchins and has been screened for its biological activity [28]. The anti-inflammatory activity of EchA, isolated from different species, has been evaluated using various in vivo and in vitro models. EchA from *Scaphchinus mirabilis* attenuated macrophage activation and neutrophil infiltration in a bleomycin (BLM)-induced scleroderma mouse model. In the same study, EchA cotreatment markedly attenuated the BLM-induced increase in the TNFα and IFNγ levels [79]. The intravenous injection of EchA in DSS-induced colitis mice significantly reduced the disease activity index (DIA), improved the colon length, and reduced the accumulation of excessive immune cells (neutrophils and macrophages) within the epithelia and mesenchymal layers of damaged colons [80]. Furthermore, EchA treatment suppressed the in vitro activation of proinflammatory M1-type macrophages and increased the production of M2-type macrophages, which abate the inflammation and initiate tissue repair [80]. EchA also reduced the level of the inflammatory cells in the aqueous humor and reduced the levels of TNFα, NF-κB, and ROS in the aqueous humor in an endotoxin-induced uveitis rat model [81]. EchA attenuated the phosphorylation of p38, ERK1/2, and JNK and thereby effectively modulated the MPAK pathway in cardiac myoblast H9c2(2-1) cells and isolated rat cardiomyocytes [82]. Histochrome® (containing 1% EchA) is a commercially available antioxidant product permitted for subconjunctival and intravenous use in Russia. Histochrome® reduced the expression of MMPs, collagen degradation, and dermal mast cell recruitment in an ultraviolet B-exposed hairless mouse model [83]. EchA treatment also reduced the inflammatory response-induced mast cell infiltration, as well as the expression of proinflammatory cytokines such as IFNγ, IL-4, and IL-13, in an acute dermatitis mouse model [84]. Spinochromes A (Figure 5: (**12**)) and B (Figure 5: (**13**)) isolated from the shells and spine of *Evechinus chloroticus* have anti-inflammatory activity in the cotton pellet granuloma rat model of chronic inflammation [85,86]. Seven major spinochromes (including EchA and Spinochromes A–D) isolated from different sea urchin species reduced TNFα production in LPS-stimulated J774A.1 macrophages [87]. In addition, the pigment isolated from the spines and shells of sea urchin *Strongylocentrotus nudus* inhibited the production of NO, IL-6, TNFα, PGE2, and 6-keto-prostaglandin F (PGF)1α in LPS-stimulated RAW264.7 macrophages [88].

### 4.2. Anti-Inflammatory Activity of Polysaccharides from Sea Urchins

Like other echinoderms, sea urchins are a rich source of bioactive polysaccharides, though few of them have been evaluated for anti-inflammatory activity. A high molecular weight sulphated polysaccharide from the eggs of the sea urchin *Paracentrotus lividus*, attenuated carrageenan-induced rat paw edema by inhibiting the production or antagonizing action of various chemical mediators such as serotonin, histamine, prostanoids, and leukotrienes [89]. In another study, gonadal polysaccharides isolated from a sea chestnut (*Anthocidaris crassispina*) reduced NO production in LPS-stimulated RAW264.7 macrophages [90].

### 4.3. Anti-Inflammatory Activity of Peptides from Sea Urchins

Centrocin 1, a peptide isolated from the green sea urchin *Strongylocentrotus droebachiensis* has shown potent anti-inflammatory activity. Centrocin 1 significantly reduced the expression of various inflammatory cytokines such as IL-12p40, IL-6, IL-1β, TNFα, and TLR 2 in *Propionibacterium acnes*-challenged monocytes [91], as well as in LPS-induced THP-1 monocytes [92]. Furthermore, Centrocin 1 attenuated proinflammatory cytokines IL-8, TNFα, and MMP-2 in the ear tissue of *Propionibacterium acnes*-induced ear swelling inflammation rat model [91]. The anti-inflammatory effect of centrocin 1 could be due to the downregulation of TLR2, which further triggers an innate immune response and the inhibition of proinflammatory cytokines [91]. Interestingly, vanadium binding protein, isolated from the blood of fresh sea urchin *Halocynthia roretzi*, reduced NO production and cytokines (COX-2, IL-1β, IL-6, and TNFα) secretion by inactivating the NF-kB and MAPK pathways in LPS-stimulated RAW264.7 macrophages [93].

### 4.4. Anti-Inflammatory Activity of Miscellaneous Compounds from Sea Urchins

**Figure 6:**
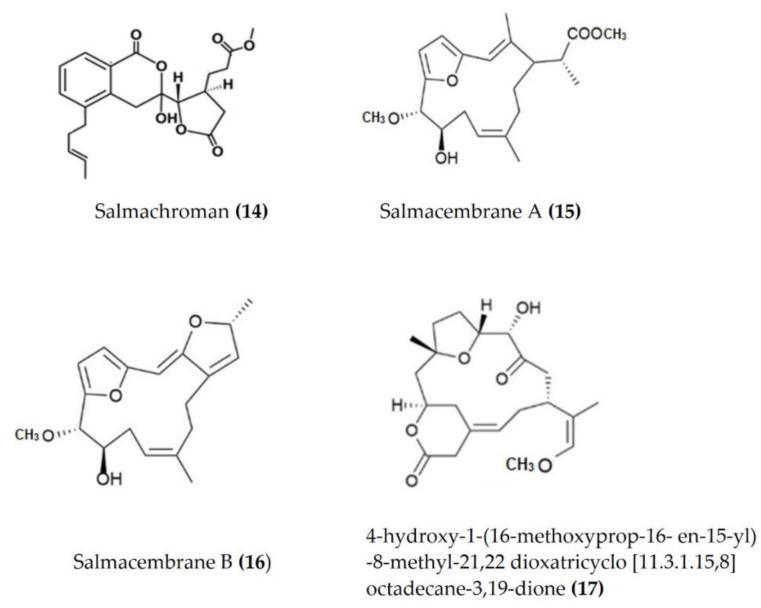
Structures of anti-inflammatory polyketides, furanocembranoids, and cembrane diterpenoids derived from sea urchins (structure (**14**) re-used with permission from [94], Taylor & Francis, 2021; structures (**15**) and (**16**) re-used with permission from reference [95], Springer Nature, 2020; structure (**17**) re-used with permission from reference [96], Springer Nature, 2020).

Several bioactive compounds such as lactones, polyketides, terpenes, and sulphonic acid derivates have been isolated from various species of sea urchins and tested for anti-inflammatory activity. Salmachroman (Figure 6: (**14**)), a polyketide isolated from *Salmacis bicolor*, possesses dual-inhibition potential against proinflammatory enzymes COX-2 and 5-LOX [94]. The polyoxygenated furanocembranoids salmacembranes A (Figure 6: (**15**)) and B (Figure 6: (**16**)) from this species also exhibited significant COX-1, COX-2, and 5-LOX inhibitory activity [95]. Several compounds have been isolated from the long-spined sea urchin *Stomopneustes variolaris* and tested for their potential to inhibit the proinflammatory eicosanoid pathway enzymes COX-2 and 5-LOX [96]. 

**Figure 7:**
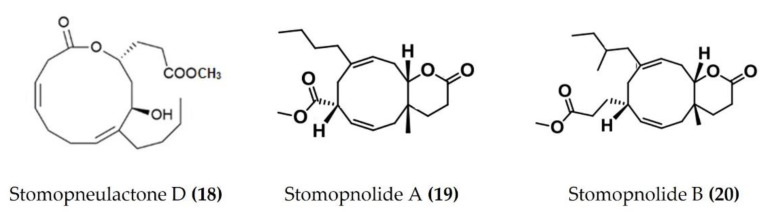
Structures of anti-inflammatory macrocyclic compounds derived from sea urchins (structure (**18**) re-used with permission from reference [97], Elsevier, 2020; structures (**19**) and (**20**) re-used with permission from reference [98], Taylor & Francis, 2021).

**Figure 8:**
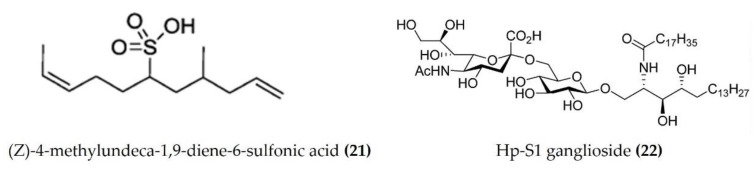
Structures of anti-inflammatory miscellaneous compounds from sea urchins (structure (**21**) re-used with permission from reference [100], Springer Nature, 2013; structure (**22**) re-used from reference [101]).

A cembrane diterpenoid, characterized as 4-hydroxy-1-(16-methoxyprop-16-en-15-yl)-8-methyl-21,22 dioxatricyclo [11.3.1.15,8], and octadecane-3,19-dione (Figure 6: (**17**)) exhibited greater inhibitory potential against inflammatory agent 5-LOX than ibuprofen. The selectivity ratio of COX-1 to COX-2 inhibition was also higher for this compound in comparison to ibuprofen [96]. The macrocyclic lactone stomopneulactone D (Figure 7: (**18**)) inhibited the generation of iNOS and inhibited COX-2 and 5-LOX in LPS-stimulated macrophages [97]. Fourteen-membered macrocyclic pyrone derivatives named stomopnolides A (Figure 7: (**19**)) and B (Figure 7: (**20**)) also showed marked 5-LOX inhibitory activity [98]. A crude lipid extract from the body wall of the sea urchin *Strongylocentrotus droebachiensis* exhibited MAPK p38, COX-1, and COX-2 inhibitory activity in LPS-stimulated human mononuclear U-937 monocytes [99]. A sulfonic acid derivative, (Z)-4-methylundeca-1,9-diene-6-sulfonic acid (Figure 8: (**21**)), isolated from the cold-water sea urchin *Brisaster latifrons* suppressed the production of proinflammatory cytokines and inflammatory responses by inactivation of the JNK/p38 MAPK and NF-κB pathways in LPS-stimulated RAW264.7 macrophages [100]. Hp-S1 ganglioside (Figure 8: (**22**)), isolated from the sperm of sea urchin *Hemicentrotus pulcherrimus* or the ovary of *Diadema setosum*, decreased the expression of iNOS and COX-2, as well as the proinflammatory cytokines TNFα, IL-1β, and IL-6. These effects of Hp-S1 were mediated through downregulating the MyD88-mediated NF-κB and JNK/p38 MAPK signaling pathways in LPS-stimulated microglial cells [101]. Ovithiol A, isolated from sea urchin *Paracentrotus lividus* eggs, decreased the expression of adhesion molecules ICAM-1 and VCAM-1 and decreased the monocyte–human umbilical vein endothelial cells interaction [102]. Phenolics, flavonoids, and proteins extracted from viscera, spines, shells, and gonads from the sea urchin *Stomopneustes variolaris* exhibit antioxidant and anti-inflammatory activities in vitro [103]. 

**Table 2:** Anti-inflammatory bioactive compounds derived from sea urchins.

Species	Bioactive Compounds/Extracts	Model	Mechanism of Anti-Inflammatory Activity	Ref.
*Scaphechinus* *mirabilis*	EchA	Bleomycin-induced scleroderma mouse model	Attenuates macrophage activation and infiltration (neutrophils), inhibits production of TNFα and IFNγ	[79]
-	EchA	DSS-induced colitis mice	Decreases DIA, improves colon length and suppresses tissue damage, suppresses macrophage activation.	[80]
-	EchA	Endotoxin-induced uveitis rat model	Reduces levels of TNFα, NF-κB antibody positive cells and ROS in aqueous humor	[81]
*-*	Histochrome^®^ (1% EchA)	UV-B exposed hairless mouse model	Reduces MMPs expression, collagen degradation and dermal inflammatory cell recruitment	[83]
*Paracentrotus* *lividus*	EchA	Stabilization of the RBCs membrane, cecal ligation and puncture model for sepsis	Potent stabilizing effect on the human RBCs, suppresses the production of IL-6 and TNFα	[104]
*Scaphechinus* *mirabilis*	Spinochromes A and B	Cotton-pellet granuloma rat model	Reduces chronic inflammation	[86]
*Echinometra mathaei*, *Diadema* *savignyi*, *Tripneustes gratilla* and *Toxopneustes* *pileolus*	Spinochromes and EchA	LPS-stimulated J774A.1 macrophages	Reduces TNFα production	[87]
*Strongylocentrotus nudus*	Spines and shells pigments	LPS-stimulated RAW264.7 macrophages	Decreases production of NO, IL-6, TNFα, PGE2 and 6-keto-PGF 1α	[88]
*Paracentrotus* *lividus*	Sulfated polysaccharide	Carrageenan-induced rat paw edema	Reduces the paw-edema	[89]
*Anthocidaris crassispina*	Gonad polysaccharide	LPS-stimulated RAW264.7 macrophages	Reduces NO production	[90]
*Strongylocentrotus* *droebachiensis*	Centrocin 1 (CEN1HC-Br)	LPS-induced THP-1 cells, Ear swelling inflammation rat model	Reduces expression of various inflammatory cytokines such as IL-12p40, IL-6, IL-1β, TNFα	[91,92]
*Salmacis bicolor*	Salmachroman	In vitro COX and 5-LOX inhibitory assays	Inhibits COX-2 and 5-LOX	[94]
*Salmacis bicolor*	Salmacembranes A and B	In vitro COX and 5-LOX inhibitory assays	Inhibits COX-1, COX-2, and 5-LOX	[95]
*Stomopneustes variolaris*	Cembrane type of diterpenoid	In vitro COX and LOX assay	Inhibits 5-LOX, high COX-1/COX-2 ratio than ibuprofen	[96]
*Stomopneustes variolaris*	Stomopneulactones D	LPS-stimulated RAW264.7 macrophages	Inhibits COX-2 and 5-LOX, reduces generation of iNOS and intracellular ROS	[97]
*Stomopneustes variolaris*	Stomopnolides A and B	In vitro 5-LOX inhibitory assays	Inhibits 5-LOX	[98]
*Strongylocentrotus droebachiensis*	Fatty acid derivatives	LPS-stimulated human mononuclear U-937 monocyte	Inhibits p38 MAPK, COX-1 and COX-2	[99]
*Brisaster latifrons*	(Z)-4-methylundeca-1,9-diene-6-sulfonic acid	LPS-stimulated RAW264.7 macrophages	Inhibits production of proinflammatory cytokines by inactivation of JNK/p38 MAPK and NF-κB pathways	[100]
*Hemicentrotus pulcherrimus* and *Diadema* *setosum*	Hp-s1 ganglioside	LPS-stimulated microglial cells	Decreases iNOS and COX-2 expression. Suppresses cytokine production. Downregulates the NF-κB and JNK/p38 MAPK signaling pathway	[101]

## 5. Anti-Inflammatory Compounds from Starfish

**Figure 9:**
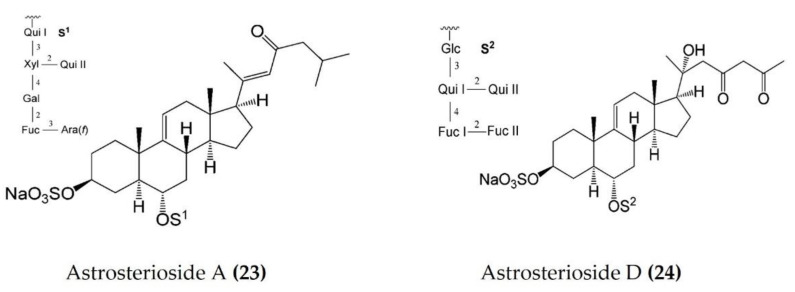
Structures of anti-inflammatory steroidal hexasaccharides derived from starfish *Astropecten monacanthus* (structures (**23**) and (**24**) re-used with permission from reference [107], ACS Publications, 2013).

**Figure 10:**
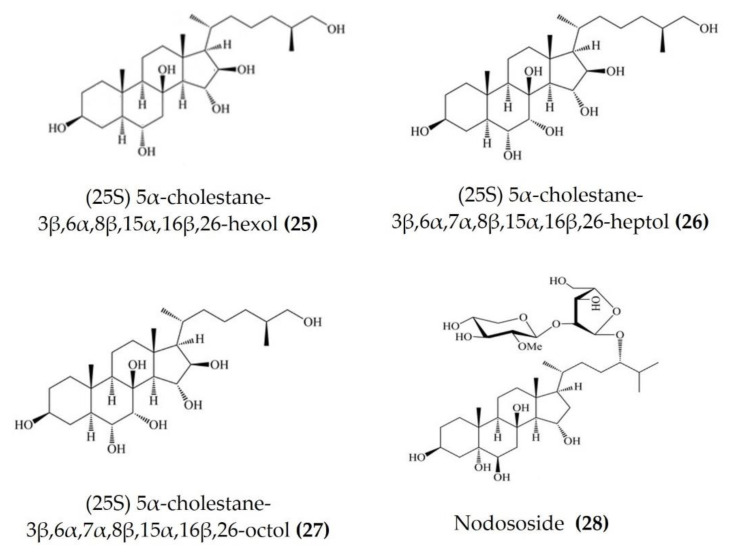
Structures of anti-inflammatory steroid derivatives derived from starfish *Protoreaster nodosus* (structures (**25**–**28**) re-used with permission from reference [110], Springer Nature, 2015).

**Figure 11:**
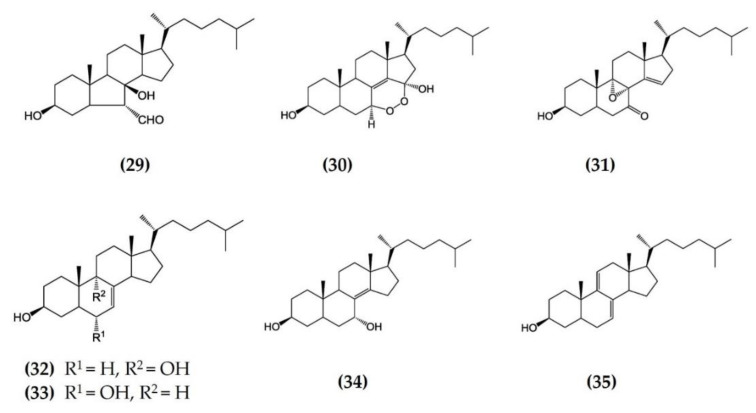
Structures of anti-inflammatory steroids derived from starfish *Astropecten polyacanthus* (structures (**29**–**35**) re-used from reference [111]).

Starfish (sea stars) are invertebrates that belong to the class Asteroidea, phylum Echinodermata. There are over 1500 species around the world, mostly inhabiting oceans, while a few occur in brackish water [105,106]. Several starfish species have been used in traditional Chinese medicine to treat various ailments such as goiters, body aches, and rheumatism [105]. In this section, we summarized the findings on the anti-inflammatory potential of various bioactive components isolated from starfish, such as glycosides, triterpenoid glycosides, steroids, and fatty acid derivatives. Several glycosides have been isolated from different species of starfish that exhibited promising preliminary anti-inflammatory activity, such as the inhibition of ROS and NO production in macrophages. Table 3 summarizes the anti-inflammatory effects displayed on LPS-stimulated RAW264.7 macrophages and bone marrow-derived dendritic cells (BMDCs) by bioactive compounds isolated from different species of starfish. Astrosterioside A (Figure 9: (**23**)) and D (Figure 9: (**24**)) and sulphated steroidal hexasaccharides isolated from starfish *Astropecten monacanthus* showed potent anti-inflammatory activity, inhibiting the secretion of proinflammatory cytokines (TNFα, IL-6, and IL-1) in LPS-stimulated BMDCs [107]. The fatty acid-rich fraction of the skin and gonads of starfish *Asterias amurensis* significantly downregulated the expression of the inflammatory mediators IL-1 β, IL-6, TNFα, iNOS, and COX-2 in LPS-stimulated RAW264.7 macrophages. This anti-inflammatory effect of fatty acids is driven through activation of the NF-κB and MAPK pathways [108]. The lipidomic profiling of spiny starfish *Marthasterias glacialis* led to the discovery of cis-11-eicosenoic and cis-11,14 eicosadienoic acids (fatty acids), as well as the unsaturated sterol ergosta-7,22-dien-3-ol. These lipids were thought to have potent anti-inflammatory activity through the reduction of ROS, NO, and proinflammatory cytokines in LPS-stimulated macrophages. Furthermore, these compounds downregulate the expression of various inflammatory genes, including iNOS, COX-2, IkBα, C/EBP homologous protein (CHOP), and NF-κB, in stimulated macrophages [109]. Oxygenated steroid derivatives (Figure 10: (**25**–**28**)) isolated from a methanol extract of the Vietnamese starfish *Protoreaster nodosus* exhibit potent anti-inflammatory activity in LPS-stimulated BMDCs, inhibiting the secretion of proinflammatory cytokines, including IL-12 p40, IL-6, and TNFα [110]. Steroids (Figure 11: (**29**–**35**)) from another Vietnamese starfish *Astropecten polyacanthus*, used as a tonic in Vietnamese ancient medicine, also exhibited potent anti-inflammatory activity when tested against LPS-stimulated BMDCs [111].

**Table 3:** Anti-inflammatory bioactive compounds derived from starfish.

Species	Bioactive Compounds/Extracts	Model	Mechanism of Anti-Inflammatory Activity	Ref.
*Astropecten* *monacanthus*	Astrosteriosides A and D	LPS-stimulated BMDCs	Inhibits secretion of proinflammatory cytokines	[107]
*Asterias* *amurensis*	Fatty acids	LPS-stimulated RAW 264.7 macrophages	Downregulates expression of inflammatory genes via NF-κB and MAPK pathways	[108]
*Marthasterias* *glacialis*	cis 11-eicosenoic and cis 11,14 eicosadienoic acids	LPS-stimulated RAW 264.7 macrophages	Downregulates inflammatory gene expression: iNOS, COX-2, IKB-α and CHOP and NF-κB	[109]
*Protoreaster nodosus*	Oxygenated steroid Derivatives	LPS-stimulated BMDCs	Inhibits secretion of proinflammatory cytokines IL-12 p40, IL-6 and TNFα	[110]
*Astropecten* *polyacanthus*	Crude extracts and steroids	LPS-stimulated BMDCs	Inhibits production of IL-12 p40, IL-6 and TNFα	[111]
*Protoreaster* *lincki*	Protolinckiosides A-D (Figure 12: (**36**–**39**))	LPS-stimulated RAW 264.7 macrophages	Reduces ROS formation and NO production	[112]
*Anthenea* *aspera*	Anthenoside O (Figure 12: (**40**))	LPS-stimulated RAW 264.7 macrophages	Reduces ROS formation and NO production	[113]
*Pentaceraster* *regulus*	Pentareguloside C (Figure 13: (**41**)) Pentareguloside D (Figure 13: (**42**)) Pentareguloside E (Figure 13: (**43**))	LPS-stimulated RAW 264.7 macrophages	Reduces ROS formation and NO production	[114]
*Acanthaster* *planci*	Plancipyrrosides A and B (Figure 14.: (**44**–**45**))	LPS-stimulated RAW 264.7 macrophages	Reduces ROS formation and NO production	[115]
*Asterina* *batheri*	Astebatheriosides B-D (Figure 15: (**46**–**48**))	LPS-stimulated BMDCs cells	Inhibits IL-12 p40 production	[116]

**Figure 12:**
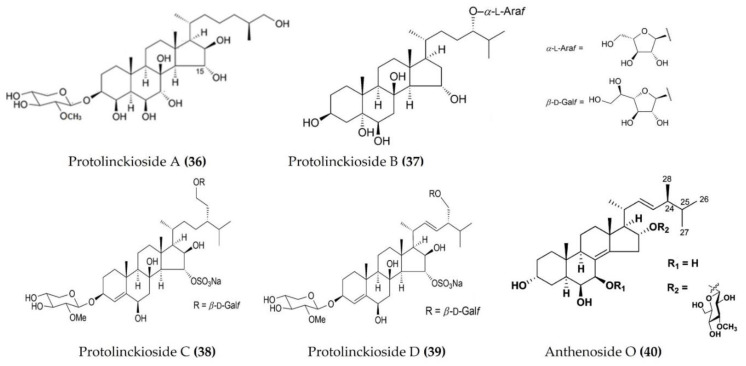
Structures of anti-inflammatory triterpenoid glycosides derived from starfish (structures (**36**–**39**) re-used with permission from reference [112], John Wiley and Sons, 2016; structure (**40**) re-used with permission from reference [113], ACS Publications, 2016).

**Figure 13:**
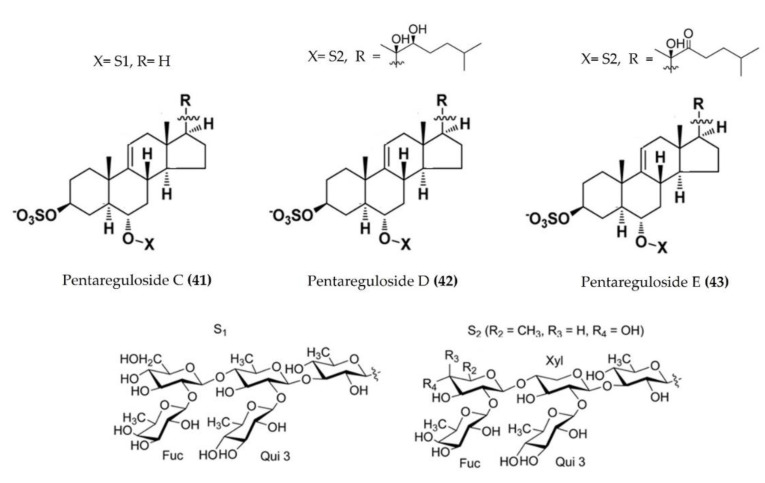
Structures of anti-inflammatory pentaregulosides (glycosides) derived from starfish *Pentaceraster regulus* (structures (**41**–**43**) re-used with permission from reference [114], ACS Publications, 2016).

**Figure 14:**
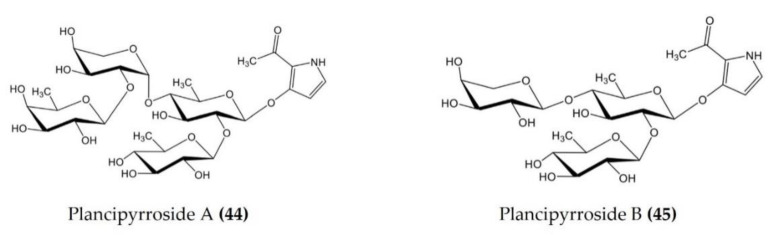
Structures of anti-inflammatory plancipyrrosides (glycosides) derived from starfish *Pentaceraster regulus* (structures (**44**–**45**) re-used from reference [115]).

**Figure 15:**
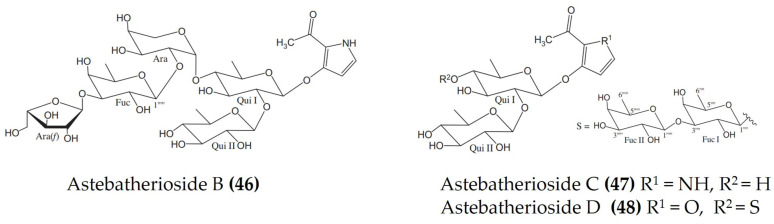
Structures of anti-inflammatory astebatheriosides (glycosides) derived from starfish *Asterina batheri* (structures (**46**–**48**) re-used with permission from reference [116], Elsevier, 2016).

## 6. Application to the Pharmaceutical Industry

The drug discovery process is a very lengthy, time-consuming, and costly process for the pharmaceutical industry, and it includes target identification, lead compound discovery, the structure–activity relationship (SAR) study, in vitro and in vivo screening, and, finally, clinical trials on large human populations. More recently, the bioinformatics approach has been employed for target identification and the discovery of lead compounds, which has significantly reduced the length of the drug discovery process [117]. Lead compounds may come from combinational chemistry, computer-aided drug design, or from natural products [118,119]. However, lead compounds often produce suboptimal biological responses and require chemical modifications to improve their efficacy and potency. The majority of drugs available clinically are derived from natural sources. Indeed, many of the anticancer small molecules available on the market are either natural products or derived from natural products [120]. The search for novel or lead compounds was previously limited to plant-based natural products but has now been expanded to marine-derived natural products as well. There have been reports of a variety of marine natural products with exploitable properties, including those that treat cancer and inflammation and neurological, immunological, and metabolic disorders [121,122,123]. The global preclinical marine pharmacology pipeline, which is still producing significant preclinical data on numerous pharmacological classes, is what provides new leads [124]. In fact, some pharmaceutical companies are focusing on marine natural product research. However, there is a general trend that anticancer drugs have received more attention, resources, and efforts in terms of pharmacological research, discovery, and development than other drugs classes, such as anti-inflammatory drugs. For example, several marine organism-derived anticancer drugs (such as vidarabine (Ara-A) for Hodgkin’s lymphoma and chronic large cell anaplastic lymphoma, cytarabine, Ara-C for acute non-lymphoblastic leukemia, and trabectedin vedotin for ovarian cancer and soft tissue sarcoma) have been approved by the FDA. Moreover, several anticancer molecules are in Phase I, II, or III clinical trials [125,126]. However, the discovery of several marine-derived anti-inflammatory molecules also has ignited the pharmaceutical industry’s interest in developing them into lead compounds for the drug discovery process [127,128]. Unfortunately, to date, no marine-derived anti-inflammatory drug has been approved by the FDA, but a few promising anti-inflammatory compounds are under various phases of clinical trials: for example, pseudopterosin A (a diterpene glycoside obtained from soft coral) and IPL-576092 (a polyhydroxylated steroid obtained from a sponge) [129]. This suggests the notable involvement of marine-derived natural products in the potential pharmaceutical industry and encourages the pursuit of new anti-inflammatory lead compound discoveries. Productive teamwork among researchers from various universities and/or institutes and the leadership of the pharmaceutical industry is required to ensure the development of future therapeutic entities that will significantly contribute to the treatment of various inflammatory disorders.

## 7. Conclusions and Research Prospects

Chronic inflammation plays a crucial role in the development of various diseases, such as inflammatory bowel disease, rheumatoid arthritis, and asthma. Controlling the progression of inflammation is a critical step in the management of these diseases. The available steroidal and nonsteroidal anti-inflammatory drugs significantly reduce chronic inflammation, but many of them can have adverse effects, such as gastrointestinal distress and liver, heart, kidney, and endocrine dysfunction, when taken long-term. In this review, the PubMed, Scopus, Web of Science, ACS, ScienceDirect, SpringerLink, and MDPI databases were searched using various combinations of keywords for publications pertaining to the anti-inflammatory potential of compounds originating from the three echinoderms: sea cucumbers, sea urchins, and starfish. Due to the immense richness and diversity of marine organisms and their natural products, it is extremely difficult to cover all of the pertinent literature, even though a broad coverage was anticipated. The major bioactive compounds isolated from sea cucumbers are fucoidan, fucosylated chondroitin sulfate, triterpenoid glycosides, small peptides, lipids, and fatty acids such as the EPA derivatives (EPA-PC and EPA-PE) sphingolipids and frondanol. Similarly, sea urchins are well-documented to produce bioactive compounds such as EchA, spine and shell pigments, polysaccharides, stomopnolides, and small peptides. Starfish also produce a diverse range of bioactive compounds, including pentaregulosides, protolinckiosides, plancipyrroside, astebatherioside, and oxygenated steroid and fatty acids. These bioactive compounds from echinoderms suppress the expression and activation of major proinflammatory cytokines such as IL-6, TNFα, IL1β, IL-10, MIP-1, etc. by the inhibition of the NF-kB and MAPK signaling pathways. Moreover, the classical COX and LOX inflammation pathway inhibitions by these compounds are also documented in this review. In addition, compounds isolated from these echinoderms also inhibit ROS generation, as well as NO production. 

Research into marine-derived anti-inflammatory lead compounds has received little consideration compared to anticancer leads; however, this is evolving very rapidly. In this review article, we presented anti-inflammatory compounds isolated from various species of sea cucumbers, sea urchins, and starfish, including their chemical structures. Many compounds, such as fucoidan, fucosylated chondroitin sulfate, eicosapentaenoic acid derivatives, and echinochrome A, have been investigated in detail for their anti-inflammatory activity and molecular mechanisms. Moreover, some novel compounds, such as glycosides from starfish, have been studied well in terms of their chemical structure and SAR with a target but only screened for preliminary anti-inflammatory activity (such as COX and 5-LOX inhibitory activity). These need further investigation to establish their molecular mechanisms. Marine pharmacology research faces many obstacles. For example, the isolation of bioactive compounds from marine organisms is extremely difficult, as they live in a complex and biodiverse environment, and it is difficult to mimic such an environment in the laboratory for their cultivation to obtain a large quantity of active substances. The future prospects in marine pharmacology should focus on following: (1) the reproduction of compounds by the chemical synthesis of established marine-derived anti-inflammatory leads to increase their production and overcome cultivation obstacles, (2) the chemical modification of existing marine-derived anti-inflammatory leads (analogs) to enhance their potency and efficacy, (3) develop lead compound libraries for large and rapid random high-throughput screening methods, (4) industry collaboration to translate preclinical leads into the clinical pipeline, and (5) establish comprehensive and efficient separation and purification techniques. The planet is gifted with vast and diverse coastlines by nature that are a treasure of bioactive compounds that have not been exploited. The scientific community should consider taking up further research to find other, potentially valuable marine drugs. 

In conclusion, this review can serve as a well-documented reference for research progress on the development of potential drugs from marine sources against various chronic inflammatory conditions.
